# Aspirin Improves Nonalcoholic Fatty Liver Disease and Atherosclerosis through Regulation of the PPAR*δ*-AMPK-PGC-1*α* Pathway in Dyslipidemic Conditions

**DOI:** 10.1155/2020/7806860

**Published:** 2020-03-19

**Authors:** Yoon-Mi Han, Yong-Jik Lee, Yoo-Na Jang, Hyun-Min Kim, Hong Seog Seo, Tae Woo Jung, Ji Hoon Jeong

**Affiliations:** ^1^Cardiovascular Center, Korea University, Guro Hospital, 148, Gurodong-ro, Guro-gu, Seoul 08308, Republic of Korea; ^2^Cellvertics Co. Ltd., Hong-Woo Building 9F, Gangnam-daero, Seocho-gu, Seoul 06621, Republic of Korea; ^3^Department of Medical Science, Korea University college of medicine (BK21 Plus KUMS Graduate Program), Main Building 6F Room 655. 73, Inchon-ro (Anam-dong 5-ga), Seongbuk-gu, Seoul 136-705, Republic of Korea; ^4^Department of Pharmacology, College of Medicine, Chung-Ang University, Seoul, Republic of Korea

## Abstract

This study is aimed at elucidating how aspirin could systemically and simultaneously normalize nonalcoholic fatty liver disease (NAFLD) and atherosclerosis through both *in vitro* and *in vivo* studies in hyperlipidemic conditions. We evaluated the effects and mechanism of aspirin on the levels of various biomarkers related to NAFLD, atherosclerosis, and oxidative phosphorylation in cells and animals of hyperlipidemic conditions. The protein levels of biomarkers (PPAR*δ*, AMPK, and PGC-1*α*) involved in oxidative phosphorylation in both the vascular endothelial and liver cells were elevated by the aspirin in hyperlipidemic condition. Also in the stimulation pathway of oxidative phosphorylation by aspirin, PPAR*δ* was a superior regulator than AMPK and PGC-1*α* in HepG2 cells. In the vascular endothelial cells, the phosphorylated endothelial nitric oxide synthase level was increased by the treatment. The protein levels of biomarkers related to lipid synthesis were decreased by the treatment in the liver cells. In rabbits administered with cholesterol diet, the levels of triglyceride, HDL-cholesterol, and alanine amino transferase in serums were ameliorated by the aspirin treatment, the levels of ATP and TNF*α* were increased or decreased, respectively, by the aspirin in liver and aorta tissues, and mannose receptor and C-C chemokine receptor type 2 levels were increased or decreased by the aspirin in spleen, respectively. The elevated levels of macrophage antigen, angiotensin II type1 receptor, and lipid accumulation were decreased in both the liver and aorta tissues in the aspirin-treated group. In conclusion, aspirin can systemically and simultaneously ameliorate NAFLD and atherosclerosis by inhibiting lipid biosynthesis and inflammation and by elevating catabolic metabolism through the activation of the PPAR*δ*-AMPK-PGC-1*α* pathway. Furthermore, aspirin may normalize atherosclerosis and NAFLD by modulating the mannose receptor and CCR2 in macrophages.

## 1. Introduction

A high-calorie diet and lack of exercise can induce metabolic diseases, such as obesity, hyperlipidemia, hypertension, cardiovascular disease, and nonalcoholic fatty liver disease (NAFLD). Atherosclerosis plays a key role in the development of cardiovascular disease, which can be fatal and is intimately correlated with the progression of NAFLD. Patients with NAFLD have low flow-mediated vasodilatation, elevated carotid-artery intimal medial thickness (a danger factor of atherosclerosis), and a high number of carotid atherosclerotic plaques [1, 2]. In addition, the main cause of death in patients with advanced NAFLD is cardiovascular disease [3]. Excessive amounts of either triglyceride or cholesterol accumulation are linked to the development of NAFLD with atherosclerosis. Triglycerides accumulate on the artery walls as well as in the liver.

However, many of the biological substrates and conditions such as insulin resistance and proinflammatory cytokines involved in the pathogenesis of NAFLD by triglyceride accumulation can also exert the effects on arteries, and its influence results in atherosclerosis [4]. Many patients with NAFLD have asymptomatic atherosclerotic lesions in their coronary artery that vary in number depending on their age [5], and the pathobiological substances related to NAFLD may accelerate these atherosclerotic lesions and lead to the development of coronary artery disease. Cholesterol can accumulate in both the arteries and liver, and NAFLD caused by hypercholesterolemia may be accompanied by atherosclerosis [6]. In our previous study, we reported that rabbits fed a high-cholesterol diet developed both early lesions of NAFLD and atherosclerosis of the aorta; furthermore, subcutaneous inflammation induced systemic inflammation and accelerated the pathogenesis of lipid-induced damage, which led to advanced lesions in both the liver and aorta [7]. Remedies for NAFLD and atherosclerosis, including common medicines, have not been developed. Therefore, discovering a common etiology and cure for atherosclerosis and NAFLD has a very important significance.

Aspirin has well-known antipyretic and analgesic effects, and these effects are achieved by the inhibition (acetylation) of cyclooxygenase 2, which converts arachidonic acid to prostaglandins (inducers of inflammation) [8].

Individual studies of aspirin treatment for atherosclerosis and NAFLD have been already reported; however, integrative and systemic preclinical research on NAFLD and atherosclerosis is lacking. Therefore, we designed and synthetically performed an investigation of the effects and mechanism of aspirin on NAFLD and atherosclerosis using *in vitro* and *in vivo* experiments to elucidate a common etiology and therapy.

## 2. Materials and Methods

### 2.1. Materials

HepG2 human liver cell line and RAW 264.7 mouse macrophage cell line were purchased from Korean Cell Line Bank (Seoul, Korea). HUVECs (human umbilical vein endothelial cells) were donated by Dr. Geum-Joon Cho (Department of Obstetrics and Gynecology, Korea University, Guro Hospital). Culture media and supplements for HepG2 and RAW 264.7 cells were obtained from Welgene Inc. (Gyeongsangbuk-do, Korea). HUVEC endothelial cell growth medium kit (EGM™-2 BulletKit™) was purchased from LONZA (Basel, Switzerland). Aspirin, cholesterol, palmitate, Compound C (dorsomorphin), Oil Red O, and GSK0660 were purchased from Sigma-Aldrich (St. Louis, USA). PRO-PREP™ protein extraction solution and prestained protein size markers were purchased from iNtRON Biotechnology. Anti-5′ adenosine monophosphate-activated protein kinase *α* (AMPK*α*) (*α* subunit total form), anti-phospho-AMPK*α* (phosphorylated at Thr172), antiendothelial nitric oxide synthase (eNOS), anti-phospho-eNOS (phosphorylated at Ser1177), and antimitochondrial transcription factor A (TFAM) primary antibodies were purchased from Cell Signaling Technology, Inc. (Danvers, MA, USA). Antiperoxisome proliferator-activated receptor gamma coactivator 1-alpha (PGC-1*α*), antiperoxisome proliferator-activated receptor delta (PPAR*δ*), anti-3-hydroxy-3-methyl-glutaryl-coenzyme A reductase (HMGCR), antinuclear factor kappa-light-chain-enhancer of activated B cells (NF-*κ*B), and antimannose receptor primary antibodies were purchased from Abcam (Cambridge, MA, USA). Anti-C-C chemokine receptor type 2 (CCR2) primary antibody was purchased from Biovison (CA, USA). Anti-fatty acid synthase (FAS) and tumor necrosis factor alpha (TNF*α*) primary antibody were purchased from Novus (Littleton, CO, USA). Antirabbit antibody, antimouse secondary antibody, and anti-*β*-actin primary antibodies were purchased from Santa Cruz Biotechnology (Santa Cruz, CA, USA). The Clarity™ Western ECL Substrate kit was purchased from Bio-Rad (Hercules, CA, USA). The X-ray film was obtained from Agfa (Mortsel, Belgium), and the developer and fixer reagents were purchased from Kodak (Rochester, NY, USA).

Enzyme-linked immunosorbent assay (ELISA) kits for adenosine triphosphate (ATP), TNF*α*, CCR2, mannose receptor, and TFAM were purchased from MyBioSource (CA, USA).

Reagents to measure the concentrations of total cholesterol, high-density lipoprotein cholesterol (HDL), and low-density lipoprotein cholesterol (LDL) were bought from Kyowa Medex Co., Ltd. (Tokyo, Japan). The oxygen consumption rate in cells was measured by the Seahorse XFp analyzer with dedicated reagents purchased from Agilent (Santa Clara, CA, USA).

### 2.2. Animal Preparation

Ten male white New Zealand rabbits (2.0~2.5 kg) were randomly divided into three groups, and they underwent experimental protocols for twelve weeks. Group 1 (three rabbits) was fed a normal control diet, group 2 (four rabbits) was fed a 1% cholesterol diet, and group 3 (three rabbits) was fed a 1% cholesterol diet with aspirin (100 mg/kg/day).

All animal experiments complied with the Korea University Animal Science Rules and Regulations, and the protocols were approved by the Korea University Institutional Animal Care and Use Committee (approval number: KUIACUC-2014-244). The white New Zealand rabbits and 1% cholesterol diet were supplied by Doo Yeol Biotech (Seoul, Korea). Rabbit normal control diet (38302AF) was obtained from Purina Korea (Gyeonggi-do, Korea). The cholesterol diet was made by Doo Yeol Biotech adding 1% cholesterol to Teklad AIN-93G Purified Diet. Ingredient compositions for 1% cholesterol and normal control diets are described in Tables 1 and 2.

### 2.3. Cell Culture

HepG2 cells and RAW 264.7 cells were cultured in high-glucose Dulbecco's Modified Eagle's Medium (DMEM) that contained 10% fetal bovine serum (FBS) and 1% antibiotic-antimycotic solution in an incubator with an atmosphere of 37°C and 5% CO_2_. The medium was replaced with fresh medium every 48–72 h. The HepG2 cells between passages 100-110 were plated in a 96-well culture plate at a density of 1 × 10^4^ cells per well or in a 6-well culture plate at 2 × 10^5^ cells per well in DMEM containing 10% FBS and 1% antibiotic-antimycotic.

The cells were cultured for 24 to 48 h in an incubator with an atmosphere of 37°C and 5% CO_2_, and the medium was changed to DMEM containing 1% FBS. Thereafter, the HepG2 cells were exposed to high-fatty acid (0.1 mM palmitate) and high-cholesterol (0.2 mM) conditions with aspirin (5 *μ*M) and the PPAR*δ* antagonist GSK0660 (50 *μ*M). The RAW 264.7 cells were exposed to high-fatty acid (30 *μ*M palmitate) and high-cholesterol (0.1 mM) conditions with aspirin (5 *μ*M) and the PPAR*δ* antagonist GSK0660 (50 *μ*M) for 24 h.

HUVECs were cultured in endothelial cell growth medium. The medium was replaced with new medium every 48–72 h. The HUVECs between passages 7 and 12 were plated in a 6-well culture plate at a density of 1 × 10^6^ cells per well in the medium. The cells were cultured for 24 to 48 h in an incubator with an atmosphere of 37°C and 5% CO_2_, and then the medium was changed to new medium. The HUVECs were then exposed to high-fatty acid (0.1 mM palmitate) and high-cholesterol (0.2 mM) conditions with aspirin (5 *μ*M) and the PPAR*δ* antagonist GSK0660 (50 *μ*M) for 24 h.

### 2.4. Western Blot Analysis

The cells were homogenized in a protein extraction solution, and protein amounts in the cell and tissue extracts were estimated by the Bradford method. The extracted proteins (10 *μ*g) were loaded onto 10% sodium dodecyl sulfate polyacrylamide electrophoresis gels. Protein blotting to nitrocellulose membranes was performed for 90 min at 100 volts. The membranes were blocked overnight in a 5% skim-milk solution or 1% bovine serum albumin in Tris-buffered saline that contained 0.05% tween 20 (TBS-T) and then washed three times for 10 min each with TBS-T. Primary antibodies were incubated with the membranes for 2 h at room temperature. The dilution conditions for the primary antibodies were as follows: PPAR*δ*, AMPK, p-AMPK (at Thr172), CCR2, p-eNOS (at Ser1177), and PGC-1*α* were 1 : 1000; TNF*α* was 1 : 500; HMGCR and NF-*κ*B were 1 : 2000; FAS was 1 : 200; and *β*-actin was 1 : 800. The membranes were washed three times for 10 min each with TBS-T and incubated with horseradish peroxidase-conjugated secondary antibodies for 1 h at room temperature. The dilution conditions for the secondary antibodies were as follows: antirabbit IgG antibodies for PPAR*δ*, AMPK, p-AMPK, and PGC-1*α* were 1 : 5000; antirabbit IgG antibodies for FAS, p-eNOS, eNOS, and FAS were 1 : 4000; antirabbit IgG antibodies for CCR2 and NF-*κ*B were 1 : 6000; antirabbit IgG antibody for TNF*α* was 1 : 3000; antigoat IgG antibody for HMGCR was 1 : 5000; and antimouse IgG antibody for *β*-actin was 1 : 6000. The membranes were then washed three times for 10 min each with TBS-T and once with TBS for 10 min. The membranes were then treated with chemiluminescent substrate and enhancer solutions. Images were obtained manually using developer and fixer reagents, and the results were analyzed by the ImageJ program.

### 2.5. The Levels of Total Cholesterol, LDL-Cholesterol, HDL-Cholesterol, Triglyceride, Alanine Aminotransferase, and Aspartate Aminotransferase in Sera

The levels of total cholesterol, LDL, HDL, triglyceride (TG), alanine aminotransferase (ALT), and aspartate aminotransferase (AST) in serum samples were estimated by the TOSHIBA TBA-2000FR (Toshiba Medical Systems Corporation, Tochigi, Japan) according to the manufacturer's instructions in the Department of Laboratory Medicine (Diagnostic Tests), Korea University, Guro Hospital (Seoul, Korea).

### 2.6. Enzyme-Linked Immunosorbent Assay (ELISA) for Adenosine Triphosphate and TNF*α* in Liver and Aorta Tissues, for Mannose Receptor and CCR2 in Spleen Tissues, and for TFAM in HepG2 Cells

The liver, aorta, and spleen tissues from the rabbits were rinsed in ice-cold phosphate-buffered saline (PBS) to remove impurities. Minced tissue of 30 mg was homogenized in 500 *μ*L of PBS. The homogenized extract was centrifuged at 5000 rpm for 10 min at 4°C, and the supernatant was transferred to another tube. To estimate the concentration of adenosine triphosphate (ATP) in the liver and aorta tissues, standard and sample solutions (100 *μ*L for each) were added to the wells and incubated at 37°C for 90 min (a blank well was set aside). After washing two times, 100 *μ*L of biotinylated rabbit ATP antibody reagent was added to each well, and the sealed plate was incubated at 37°C for 60 min. After washing three times, 100 *μ*L of enzyme-conjugate reagent was added to each well, except for the blank well, and the sealed plate was incubated at 37°C for 60 min. After washing five times, 100 *μ*L of color reagent was added to each well including the blank well, and the sealed plate was incubated in dark conditions for 10 min at 37°C. Then, 100 *μ*L of stop solution was added to the individual wells, and the absorbance at 450 nm was estimated with an ELISA reader. The concentration of ATP in the liver and aorta tissues was determined by interpolation from a standard curve prepared with standard samples supplied by the manufacturer and expressed in ng/mL.

To estimate the TNF*α* level in liver and aorta tissues, standard and sample solutions (100 *μ*L for each) were added to the wells and incubated at 37°C for 90 min. After removing the liquid out of each well, 100 *μ*L of biotinylated rabbit TNF*α* detection antibody reagent was added to each well, and the sealed plate was incubated at 37°C for 60 min. After washing three times, 100 *μ*L of horseradish peroxidase- (HRP-) conjugate solution was added to each well and the sealed plate was incubated at 37°C for 30 min. After washing five times, 90 *μ*L of substrate reagent was added to each well and the sealed plate was incubated in dark conditions for 15 min at 37°C. Then, 50 *μ*L of stop solution was added to the individual wells, and the absorbance at 450 nm was estimated with an ELISA reader. The concentration of TNF*α* in the liver and aorta tissues was determined by interpolation from a standard curve prepared with standard samples supplied by the manufacturer and expressed in ng/mL.

To estimate the concentration of mannose receptor in the spleen tissues, standard and sample solutions (100 *μ*L for each) were added to the wells and incubated at 37°C for 90 min (a blank well was set aside). After washing two times, 100 *μ*L of biotinylated rabbit mannose receptor antibody reagent was added to each well, and the sealed plate was incubated at 37°C for 60 min. After washing three times, 100 *μ*L of enzyme-conjugate reagent was added to each well, except for the blank well, and the sealed plate was incubated at 37°C for 30 min. After washing five times, 100 *μ*L of color reagent was added to each well including the blank well, and the sealed plate was incubated in dark conditions for 10~20 min at 37°C. Then, 100 *μ*L of color reagent C was added to the individual wells, and the absorbance at 450 nm was estimated with an ELISA reader. The concentration of mannose receptor in the spleen tissues was determined by interpolation from a standard curve prepared with standard samples supplied by the manufacturer and expressed in mg/dL.

To estimate the concentration of CCR2 in spleen tissues, standard and sample solutions (100 *μ*L for each) were added to the wells and 10 *μ*L of balance solution was added to sample wells. 50 *μ*L of conjugate reagent was added to each well (not blank control well), well mixed, and incubated at 37°C for 60 min. After washing five times, 50 *μ*L substrate A and 50 *μ*L substrate B were added to each well (including blank control well), sealed, and incubated for 15~20 min at 37°C in the dark condition.

Then, 50 *μ*L of stop solution was added to the individual wells, and the absorbance at 450 nm was estimated with an ELISA reader. The concentration of CCR2 in the spleen tissues was determined by interpolation from a standard curve prepared with standard samples supplied by the manufacturer and expressed in mg/dL.

To estimate the concentration of TFAM in HepG2 cell extracts, standard and sample solutions (100 *μ*L for each) were added to the wells, well mixed, and incubated at 37°C for 60 min. After removing the liquid of each well, 100 *μ*L of detection reagent A was added to each well, well mixed, and incubated at 37°C for 60 min. After removing the liquid of each well and washing three times, 100 *μ*L of detection reagent B was added to each well, well mixed, and incubated at 37°C for 30 min. After removing the liquid of each well and washing five times, 90 *μ*L of substrate solution was added to each well, well mixed, and incubated at 37°C for 10 min. 50 *μ*L of stop solution was added to each well, and the absorbance at 450 nm was estimated with an ELISA reader. The concentration of TFAM in the HepG2 cells extracts was determined by interpolation from a standard curve prepared with standard samples supplied by the manufacturer and expressed in ng/mL.

### 2.7. Immunohistochemistry (IHC)

Frozen-sectioned tissue slides were fixed in 4% formaldehyde solution for 15 min followed by ice-cold methanol for 5 min. The fixed slides were incubated in a 0.3% H_2_O_2_ solution for 10 min, washed, and then blocked using a normal serum solution for 20 min. The blocked slides were treated with primary antibody for 1 h and then washed with PBS. The slides were incubated with secondary antibody for 30 min and then washed with PBS. The slides were incubated with the premixed VECTASTAIN ABC solution for 30 min, washed with PBS, and then incubated with 3,3′-diaminobenzidine (DAB) substrate solution until color development. After DAB colorization, the slides were washed with tap water for 5 min, counterstained with hematoxylin, washed again with tap water, air-dried, and finally mounted.

### 2.8. Immunocytochemistry (ICC)

The cells were fixed with methanol for 15 min at 20°C. Peroxidase removal was performed as follows: 0.3% H_2_O_2_ solution for 5 min, washed, and blocked using normal serum solution for 20 min. The cells were then incubated with primary antibody for 1 h and washed with PBS. The slides were incubated with secondary antibody for 30 min and washed with PBS. The slides were then incubated with premixed VECTASTAIN ABC solution for 30 min. The slides were washed with TBS-T and then incubated with DAB substrate solution until color development. After color development, the slides were washed with tap water for 5 min, counterstained with hematoxylin, washed again with tap water, air-dried, and finally mounted.

### 2.9. Oil Red O Staining

The cell culture medium was completely removed, and the cells were rinsed with PBS. The PBS was aspirated completely, 10% formaldehyde solution was added to the cells, and the cells were incubated for 30 min at room temperature. After removing the formaldehyde solution, the fixed cells were gently washed with PBS. The Oil Red O solution was added to the wells, and the samples were incubated for 60 min at room temperature. The cells were washed with PBS and photographed using an optical microscope (Olympus BX51). The Oil Red O dye was eluted by 100% isopropyl alcohol, and the absorbance at 530 nm was measured using the SpectraMax Plus 384 Microplate Reader (Molecular Devices LLC., Sunnyvale, CA, USA).

For frozen-sectioned tissue slides, the slides were fixed in 4% formaldehyde solution for 30 min, washed with tap water for 5 min, and washed with 60% isopropyl alcohol for 2 min. After staining with 60% Oil Red O working solution, the slides were washed with tap water for 3 min. The slides were then counterstained with hematoxylin solution for 1 min and washed with tap water until the water flowed clear. The slides were air-dried, mounted, and then observed and photographed using an optical microscope.

### 2.10. Oxygen Consumption Rate Analysis

The oxygen consumption rate in the HepG2 cells and HUVECs was analyzed by the Seahorse XFp system (Agilent, Santa Clara, CA, USA) according to the manufacturer's protocol. The cells were plated at 1.5 × 10^4^ cells per well. After the cells had time to settle, the drug was added to the media, and the cells were incubated overnight in an incubator with an atmosphere of 37°C and 5% CO_2_. A sensor cartridge+utility plate that contained calibrant was incubated overnight in a non-CO_2_ incubator at 37°C. On the day of the analysis, assay media were prepared similar to culture media (25 mM glucose and 4 mM L-glutamine), and the pH was adjusted to 7.4. The XFp miniplate was washed twice with the assay media, and the assay media (a final volume of 180 *μ*L) were added to the cells. The XFp miniplate was allowed to equilibrate in a non-CO_2_ incubator at 37°C for 60 min prior to assay initiation. Oligomycin, carbonyl cyanide-4-(trifluoromethoxy) phenylhydrazone, and antimycinA/rotenone were separately injected into each drug port in the sensor cartridge+utility plate and incubated in a non-CO_2_ incubator for 10 min.

### 2.11. Statistical Analysis

The data are presented as the mean ± standard error of the means (SEM). Statistically significant differences between two groups were calculated by the unpaired *t*-test, and the one-way ANOVA test was used to compare the means of three or more groups. The correlation among results of biomarkers was analyzed by the Spearman correlation coefficient. A value <0.05 was considered statistically significant.

## 3. Results

### 3.1. Aspirin Treatment Increased the Protein Levels of PPAR*δ*, p-AMPK, PGC-1*α*, and p-eNOS in HUVECs Treated with High Concentrations of Palmitate and Cholesterol; However, It Decreased AT1R in the Same Condition. Aspirin Treatment Elevated the Oxygen Consumption Rate in HUVECs

PPAR*δ*, AMPK, and PGC-1*α* function as essential regulators of cell energy metabolism. PPAR*δ* is a nuclear transcription factor that is ubiquitously expressed in many tissues, such as the liver, heart, colon, and skeletal muscle and ameliorates several chronic diseases, including diabetes, obesity, atherosclerosis, and cancer [9, 10]. In addition, the activation of PPAR*δ* by its agonist stimulates fatty acid oxidation and protects the mitochondria in pancreatic *β*-cells [11]. AMPK maintains cellular energy homeostasis and activates the catabolic metabolism of glucose and lipids [12–14]. PGC-1*α* is an essential transcriptional coactivator and regulates the genes involved in catabolic metabolism that produce ATP in heart and skeletal muscle [15, 16]. Blood pressure is directly regulated by AT1R and nitric oxide in the blood vessels. Nitric oxide is the main vasodilating factor produced by eNOS in vascular endothelium, and it plays a crucial role in the maintenance of vascular homoeostasis and prevention of atherothrombotic events [17]. Furthermore, the expressions of AT1R and eNOS are regulated by AMPK and PGC-1*α* [18–20].

**Figure 1:**
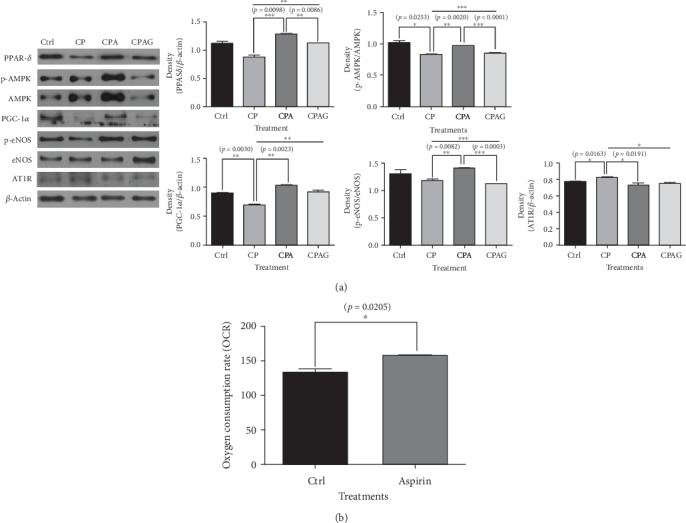
Protein levels of PPAR*δ*, p-AMPK, PGC-1*α*, AT1R, and p-eNOS in HUVECs that were treated with high concentrations of palmitate, cholesterol, and aspirin. The oxygen consumption rate in HUVECs treated with aspirin. (a) (A-1, A-2, A-3, A-4) The protein levels of PPAR*δ* (A-1), p-AMPK (A-2), PGC-1*α* (A-3), and p-eNOS (A-4) were higher in the CPA group, but they were reversed by a PPAR*δ* antagonist. (A-5) The protein level of AT1R was higher in the CP group, but it was decreased in the CPA group. (b) Aspirin treatment increased the oxygen consumption rate in HUVECs. The results are expressed as means ± SEM (*N* = 3). Values were statistically analyzed by unpaired *t*-test and one-way ANOVA. An upper line on the three bars means one-way ANOVA analysis. All experiments were repeated three and over times. Meaning of indications: Ctrl is an untreated control group, CP is a cholesterol and palmitate-treated group, CPA is a cholesterol, palmitate, and aspirin-treated group, and CPAG is a cholesterol, palmitate, aspirin, and GSK0660-treated group. ^∗^*p* < 0.05, ^∗∗^*p* < 0.01, ^∗∗∗^*p* < 0.001.

**Figure 2:**
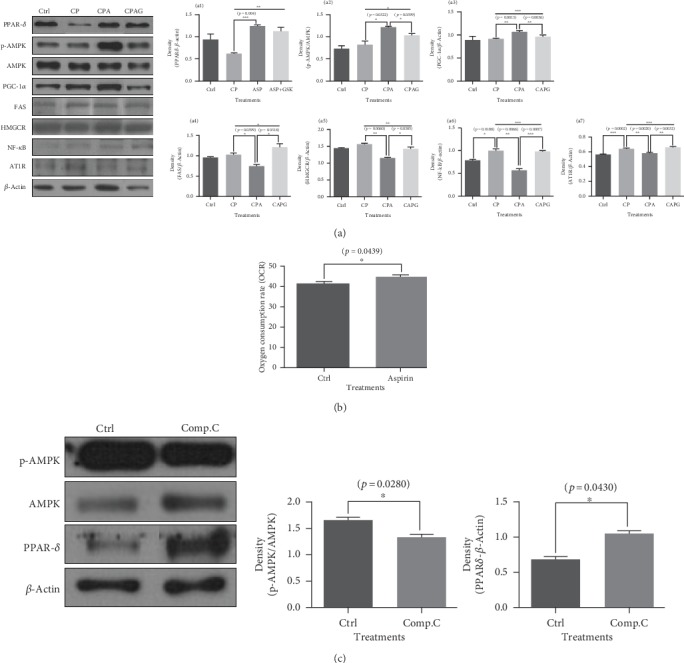
Protein levels of PPAR*δ*, p-AMPK, PGC-1*α*, FAS, HMGCR, NF-*κ*B, and AT1R in HepG2 cells that were treated with high concentrations of palmitate, cholesterol, and aspirin. The oxygen consumption rate in HepG2 cells treated with aspirin. The effects of AMPK antagonist on p-AMPK and PPAR*δ* protein levels in HepG2 cells. (a) (A-1, A-2, A-3) The protein levels of PPAR*δ* (A-1), p-AMPK (A-2), and PGC-1*α* (A-3) were higher in the CPA group than those of the CP group; however, the effects of aspirin were reversed by treatment with a PPAR*δ* antagonist. (A-4, A-5, A-6, A-7) The protein levels of FAS (A-4), HMGCR (A-5), NF-*κ*B (A-6), and AT1R (A-7) were lower in the CPA group than those of the CP group; however, the effects of aspirin were reversed by treatment with a PPAR*δ* antagonist. (b) The oxygen consumption rate was increased by aspirin treatment. (c) AMPK antagonist, Compound C, decreased only the protein expression of p-AMPK. The results are expressed as means ± SEM (*N* = 3). Values were statistically analyzed by unpaired *t*-test and one-way ANOVA. An upper line on the three bars means one-way ANOVA analysis. All experiments were repeated three and over times. Meaning of indications: Ctrl is an untreated control group, CP is a cholesterol and palmitate-treated group, CPA is a cholesterol, palmitate, and aspirin-treated group, and CPAG is a cholesterol, palmitate, aspirin, and GSK0660-treated group. ^∗^*p* < 0.05, ^∗∗^*p* < 0.01, ^∗∗∗^*p* < 0.001.

**Figure 3:**
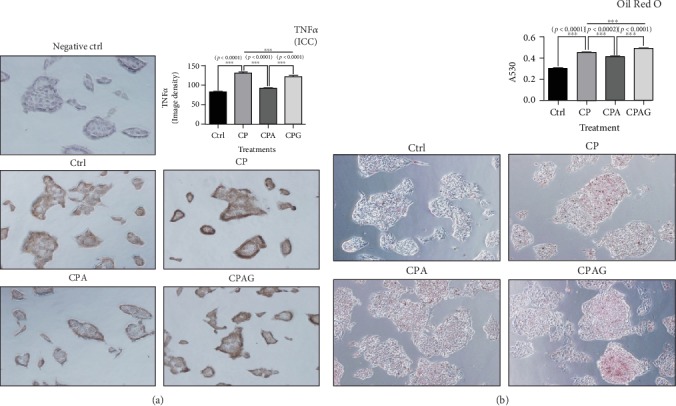
Immunocytochemistry for TNF*α* and Oil Red O staining in HepG2 cells treated with high concentrations of palmitate, cholesterol, and aspirin. (a) The elevated TNF*α* protein expression in the CP group was decreased by aspirin; however, the effect of aspirin was reversed by treatment with a PPAR*δ* antagonist. (b) Lipid accumulation increased in the CP group was decreased by aspirin treatment; however, the effect of aspirin was offset by treatment with a PPAR*δ* antagonist. Images were taken at ×200 magnification. The results are expressed as means ± SEM (*N* = 3 or 6). Values were statistically analyzed by unpaired *t*-test and one-way ANOVA. An upper line on the three bars means one-way ANOVA analysis. All experiments were repeated three and over times. Meaning of indications: Ctrl is an untreated control group, CP is a cholesterol and palmitate-treated group, CPA is a cholesterol, palmitate, and aspirin-treated group, and CPAG is a cholesterol, palmitate, aspirin, and GSK0660-treated group. ^∗∗∗^*p* < 0.001.

**Figure 4:**
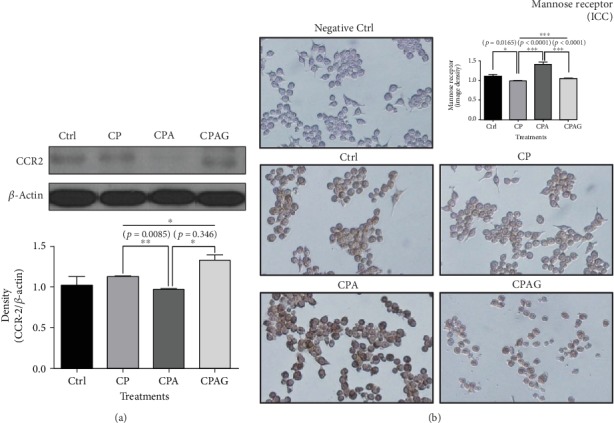
CCR2 western blot analysis and immunocytochemistry of mannose receptor in RAW 264.7 cells treated with high concentrations of palmitate, cholesterol, and aspirin. (a) The protein level of CCR2 was lower in the CPA group than that of the CP group; however, the effect of aspirin was reversed by treatment with a PPAR*δ* antagonist. (b) The protein expression of mannose receptor was increased by aspirin treatment. But the effect of aspirin was offset by a PPAR*δ* antagonist. Images were taken at ×200 magnification. The results are expressed as means ± SEM (*N* = 3). Values were statistically analyzed by unpaired *t*-test and one-way ANOVA. An upper line on the three bars means one-way ANOVA analysis. All experiments were repeated three and over times. Meaning of indications: Ctrl is an untreated control group, CP is a cholesterol and palmitate-treated group, CPA is a cholesterol, palmitate, and aspirin-treated group, and CPAG is a cholesterol, palmitate, aspirin, and GSK0660-treated group. ^∗^*p* < 0.05, ^∗∗^*p* < 0.01, ^∗∗∗^*p* < 0.001.

**Figure 5:**
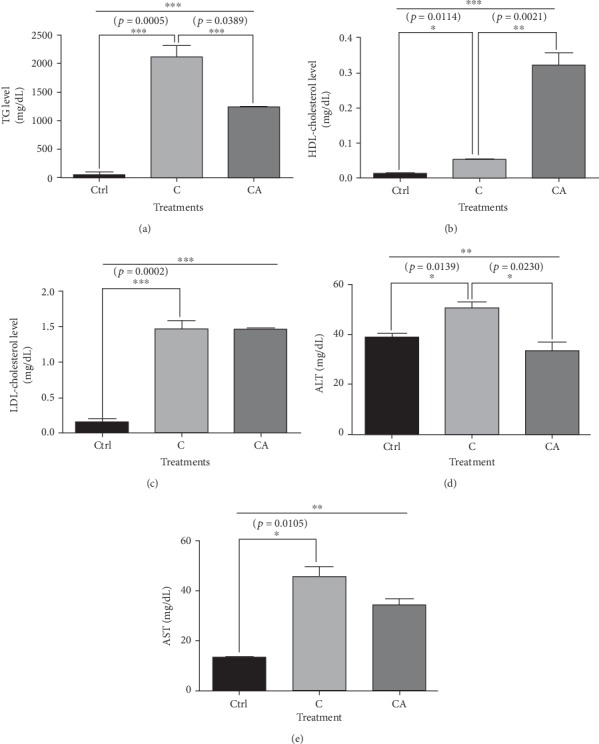
Concentrations of triglyceride (TG), high-density lipoprotein cholesterol (HDL), and low-density lipoprotein cholesterol (LDL) in sera from rabbits administered a cholesterol diet and aspirin. (a) TG level was higher in the cholesterol-diet rabbits than that of the control rabbits, and it was lower in the aspirin-administered rabbits. (b) HDL-cholesterol level was more significantly elevated in the aspirin-administered rabbits than that of the cholesterol-diet rabbits without aspirin. (c) LDL-cholesterol level was higher in the cholesterol-diet rabbits than that of the control rabbits, and it was not decreased in the aspirin-administered rabbits. (D, E) ALT and AST levels were higher in the cholesterol-diet rabbits than those of the control rabbits; however, they were decreased in the aspirin-administered rabbits. The results are expressed as means ± SEM (*N* = 3 or 4). Values were statistically analyzed by unpaired *t*-test or one-way ANOVA. An upper line on the three bars means one-way ANOVA analysis. All experiments were repeated three and over times. Meaning of indications: Ctrl means normal control rabbits administered with normal, C means rabbits fed with 1% cholesterol diet, and CA means rabbits administered with 1% cholesterol diet plus aspirin of 100 mg/kg/day. ^∗^*p* < 0.05, ^∗∗^*p* < 0.01, ^∗∗∗^*p* < 0.001.

The results of our *in vitro* experiments on the effect of aspirin on atherosclerosis showed elevated protein levels of PPAR*δ*, AMPK, PGC-1*α,* and eNOS in the aspirin-treated group compared to the levels in the high-fatty acid and high-cholesterol group without aspirin; however, these increases were reversed by treatment with the PPAR*δ* antagonist GSK0660 (Figure 1(a), A-1–A-4). The protein expression of AT1R increased in the high-fatty acid and high-cholesterol group without aspirin, but it decreased in the aspirin-treated group (Figure 1, A-5).

ATP is mainly produced from oxidative phosphorylation via the electron transport chain using oxygen as the final receptor of electrons in aerobic eukaryotes. Therefore, the oxygen consumption rate directly presents the extent to which catabolic metabolism produces ATP. In our study, a 5 *μ*M aspirin treatment elevated the oxygen consumption rate in HUVECs (Figure 1(b)).

### 3.2. Aspirin Treatment Increased the Protein Levels of PPAR*δ*, p-AMPK, and PGC-1*α* in HepG2 Cells Treated with High Concentrations of Palmitate and Cholesterol; However, Aspirin Treatment Decreased the Protein Levels of FAS, HMGCR, NF-*κ*B, and AT1R. Aspirin Treatment Elevated the Oxygen Consumption Rate in HepG2 Cells

FAS and HMGCR are involved in lipid and cholesterol synthesis in each. Lipid peroxidation products (e.g., 4-hydroxynonenal) and reactive oxygen species (e.g., H_2_O_2_) activate NF-*κ*B, and then NF-*κ*B upregulates proinflammatory cytokines such as TNF*α* [21, 22]. Therefore, the expressions of FAS, HMGCR, NF-*κ*B, and TNF*α* may be intimately related to the pathogenesis of NAFLD.

**Figure 6:**
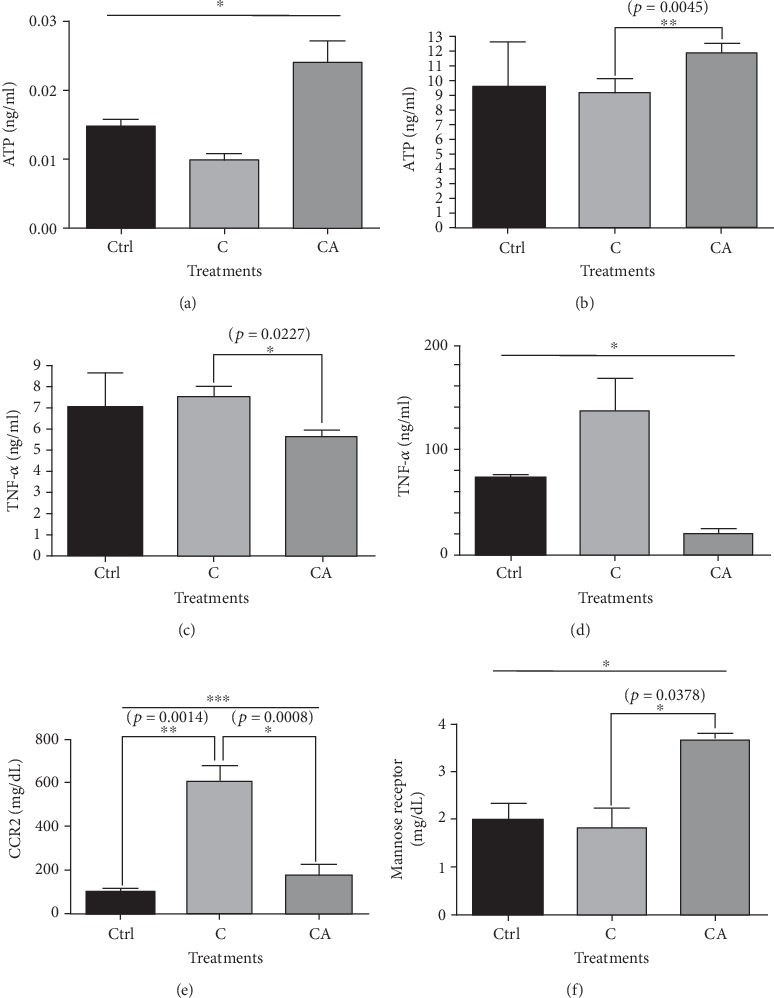
Concentrations of ATP and TNF*α* in the aorta and liver, and concentrations of mannose receptor and CCR2 in spleen from rabbits administered a cholesterol diet and aspirin. (a) ATP concentration in the aorta showed a tendency to increase in the aspirin-administered group than that of the cholesterol-diet group without aspirin. (b) ATP concentration in the liver was higher in the aspirin-administered group than that of the cholesterol-diet group without aspirin. (c) TNF*α* concentration in the aorta was lower in the aspirin-administered group than that of the cholesterol-diet group without aspirin. (d) TNF*α* concentration in the liver showed an increasing trend in the cholesterol-diet group than that of the control group; however, it was lower in the aspirin-administered group than that of the cholesterol-diet group without aspirin. (e) CCR2 concentration in the spleen was higher in the cholesterol-diet group than that of the control group; however, it was lower in the aspirin-administered group. (f) Mannose receptor concentration in the spleen was higher in the aspirin-administered group than that of the cholesterol-diet group without aspirin. The results are expressed as means ± SEM (*N* = 3 or 4). Values were statistically analyzed by unpaired *t*-test or one-way ANOVA. An upper line on the three bars means one-way ANOVA analysis. All experiments were repeated three and over times. Meaning of indications: Ctrl means normal control rabbits administered with normal, C means rabbits fed with 1% cholesterol diet, and CA means rabbits administered with 1% cholesterol diet plus aspirin of 100 mg/kg/day. ^∗^*p* < 0.05, ^∗∗^*p* < 0.01, ^∗∗∗^*p* < 0.001.

**Figure 7:**
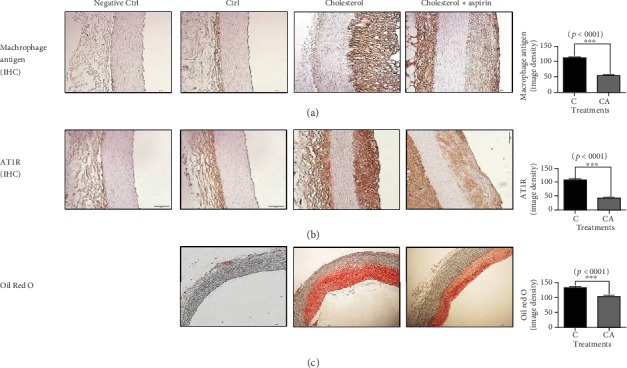
Immunohistochemistry for macrophage antigen and AT1R and Oil Red O staining in frozen-sectioned aorta tissue slides from rabbits administered a cholesterol diet and aspirin. (a, b) The protein expressions for macrophage antigen and AT1R in aorta tissues were extremely increased in cholesterol diet group compared to control; however, they were decreased in aspirin-administered group. (c) The lipid accumulation in aorta tissues of cholesterol diet group was elevated compared to control; however, the accumulation was lowered in aspirin-administered group. Magnification is 200 times for images of immunohistochemistry and 40 times for images of Oil Red O staining. Densities for images were analyzed with the ImageJ program. The results of image density are expressed as means ± SEM (*N* = 10). Values were statistically analyzed by unpaired *t*-test or one-way ANOVA. An upper line on the three bars means one-way ANOVA analysis. All experiments were repeated three and over times. Meaning of indications: C means rabbits fed with 1% cholesterol diet and CA means rabbits administered with 1% cholesterol diet plus aspirin of 100 mg/kg/day. ^∗∗∗^*p* < 0.001.

The protein levels of PPAR*δ*, AMPK, and PGC-1*α* were elevated in the aspirin-treated group compared to the levels in the high-fatty acid and high-cholesterol group without aspirin; however, the protein levels of FAS, HMGCR, NF-*κ*B, and AT1R decreased with aspirin treatment. The effects of aspirin were reversed by treatment with the PPAR*δ* antagonist GSK0660 (Figure 2, A-1–A-7). The oxygen consumption rate in the HepG2 cells was upregulated by a 5 *μ*M aspirin treatment (Figure 2(b)). AMPK antagonist, Compound C, decreased AMPK protein expression, but it rather increased the protein level of PPAR*δ* (Figure 2(c)).

### 3.3. Aspirin Treatment Decreased TNF*α* Protein Expression in HepG2 Cells Treated with High Concentrations of Palmitate and Cholesterol; in Addition, Aspirin Treatment Reduced Lipid Accumulation in the Same Cells under Identical Treatment Conditions

The protein expression of TNF*α* was higher in the palmitate and cholesterol-treated group compared than that of the control group in HepG2 cells, but its expression decreased with aspirin treatment (Figure 3(a)).

The lipid accumulation in the HepG2 cells that were treated with palmitate and cholesterol was higher than that of the control group; however, the lipid content decreased with aspirin treatment (Figure 3(b)).

The normalized TNF*α* expression and lipid profile of the HepG2 cells treated with aspirin increased when treated with a PPAR*δ* antagonist (Figures 3(a) and 3(b)).

### 3.4. Aspirin Treatment Decreased the Protein Level of CCR2 in RAW 264.7 Cells Treated with High Concentrations of Palmitate and Cholesterol; However, Aspirin Treatment Increased the Protein Expression of Mannose Receptor in the Same Cells under Identical Treatment Conditions

CCR2 is involved in monocyte chemotaxis and is important in the pathogenesis of NAFLD. In addition, CCR2 is related to the occurrence of atherosclerosis [23, 24]. The CCR2 protein level of the RAW 264.7 cells in the palmitate and cholesterol-treated group was higher than that of the control group; however, the CCR2 level was lower in the aspirin-treated group than that of the palmitate and cholesterol-treated group (Figure 4(a)).

Mannose receptor (Cluster of Differentiation 206, CD206) is a representative marker of noninflammatory macrophage type 2 (M2) and is known to promote tissue repair [25]. Based on the results of a previous study that showed inflammatory macrophage type 1 (M1) increased in NAFLD [26], we examined whether RAW 264.7 cells changed to M2, which is a noninflammatory state, with aspirin treatment. Mannose receptor expression was lower in the palmitate and cholesterol-treated group than that of the control group, but it was higher in the aspirin-treated group than that of the palmitate and cholesterol-treated group (Figure 4(b)). The effects of aspirin treatment on CCR2 and mannose receptor levels in our study were reversed by treatment with the PPAR*δ* antagonist GSK0660 (Figures 4(a) and 4(b)).

### 3.5. Aspirin Ameliorated Diet Intake Amount and Body Weight Decreased by Cholesterol Diet

The diet intake amount containing cholesterol was smaller than that in the control group during the experimental period in general. Especially, rabbits of cholesterol-diet group ate very small diet compared to the control group for 1~4 weeks, and then they ate well cholesterol diet for 5~9 weeks. But, in the late period (10~13 weeks) of experiment, the cholesterol diet consumption amount was decreased again in cholesterol diet fed group ([Supplementary-material supplementary-material-1]). And the body weight of rabbits in cholesterol fed group was lower than control ([Supplementary-material supplementary-material-1]).

But the decreased diet intake amount and weight loss by cholesterol diet was ameliorated via the treatment of aspirin ([Supplementary-material supplementary-material-1]).

### 3.6. Aspirin Treatment Decreased the Elevated Levels of TG, ALT, and AST in the Sera of Rabbits Fed a Cholesterol Diet; However, Aspirin Treatment Increased HDL Level

The elevated concentration of TG in the cholesterol-diet group was lowered with aspirin treatment compared to that of the control group (Figure 5(a)). The concentration of HDL increased over 6-times in the aspirin-treated group compared to that of the cholesterol-diet group (Figure 5(b)); however, LDL was not affected by aspirin treatment (Figure 5(c)). The elevated ALT and AST levels in the cholesterol-diet rabbits compared to those of the control group decreased or showed a tendency to decrease in the aspirin plus cholesterol-diet group, respectively (Figures 5(d) and 5(e)).

### 3.7. Aspirin Administration Increased ATP Levels in the Aorta and Liver of Rabbits Fed a Cholesterol Diet; However, Aspirin Administration Decreased the Concentration of TNF*α* in the Aorta and Liver. Aspirin Administration Decreased CCR2 Levels in the Spleen but Increased Mannose Receptor Levels in the Rabbits Fed a Cholesterol Diet

ATP concentration in the aorta showed a decreasing trend in the rabbits fed a cholesterol diet; however, the concentration showed an increasing trend in the rabbits fed a cholesterol diet with aspirin (Figure 6(a)). ATP concentration in the liver increased in the rabbits fed a cholesterol diet plus aspirin compared to the concentration in the rabbits fed a cholesterol-only diet (Figure 6(b)). TNF*α* concentration in the aorta decreased in the rabbits fed a cholesterol diet plus aspirin compared to the concentration in the rabbits fed a cholesterol-only diet (Figure 6(c)). TNF*α* concentration in the liver showed an increasing trend in the rabbits fed a cholesterol-only diet compared to the concentration in the control group; however, the concentration showed a decreasing tendency with aspirin administration (Figure 6(d)).

CCR2 concentration in the spleen increased in the rabbits fed a cholesterol-only diet compared to the concentration in the control group; however, the concentration decreased with aspirin administration (Figure 6(e)).

Mannose receptor concentration in the spleen increased in the rabbits fed a cholesterol diet plus aspirin compared to the concentration in the rabbits fed a cholesterol-only diet (Figure 6(f)).

### 3.8. In Both the Aorta and Liver Tissues from the Rabbits Fed a Cholesterol Diet, Aspirin Administration Decreased Lipid Accumulation and the Protein Expressions of Macrophage Antigen and AT1R

**Figure 8:**
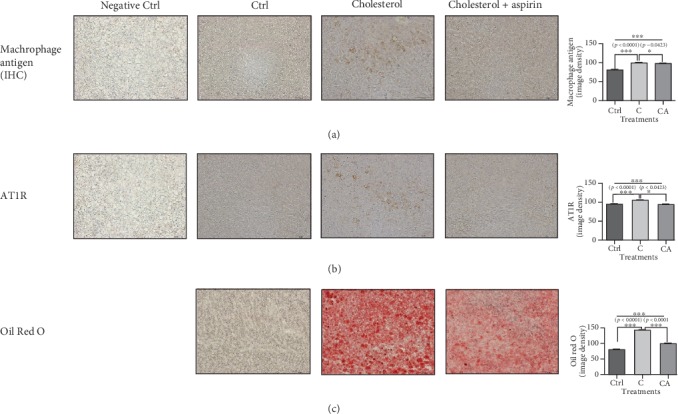
Immunohistochemistry for macrophage antigen and AT1R and Oil Red O staining in frozen-sectioned liver tissue slides from rabbits administered a cholesterol diet and aspirin. (a, b) The protein expressions for macrophage antigen and AT1R in liver tissues were increased in cholesterol-diet group compared to control; however, they were decreased in aspirin-administered group. (c) The lipid accumulation in liver tissues of cholesterol-diet group was elevated compared to control; however, the accumulation was lowered in aspirin-administered group. Magnification is 200 times. Densities for images were analyzed with the ImageJ program. The results of image density are expressed as means ± SEM (*N* = 10). Values were statistically analyzed by unpaired *t*-test or one-way ANOVA. An upper line on the three bars means one-way ANOVA analysis. All experiments were repeated three and over times. Meaning of indications: Ctrl means normal control rabbits administered with normal, cholesterol means rabbits fed with 1% cholesterol diet, and cholesterol+aspirin means rabbits administered with 1% cholesterol diet plus aspirin of 100 mg/kg/day. ^∗^*p* < 0.05, ^∗∗^*p* < 0.01, ^∗∗∗^*p* < 0.001.

Previous studies have reported that the protein expressions of MCP-1, TNF, and AT1R are elevated and play a significant role in atherosclerosis [27–29]. In our rabbit aorta study, lipid accumulation and the protein expressions of macrophage antigen and AT1R were decreased in the aspirin-administered rabbits compared to those in the cholesterol-diet rabbits without aspirin (Figure 7). Likewise, lipid accumulation and the protein expressions for macrophage antigen and AT1R in the liver were lower in the aspirin-treated group compared to those in the cholesterol-diet group without aspirin (Figure 8).

The results of macrophage antigen, AT1R, and Oil Red O staining in liver and aorta showed correlations in general ([Supplementary-material supplementary-material-1]).

## 4. Discussion

Atherosclerosis and NAFLD are pathologically related, and the efficiency of aspirin in these two diseases has been proven in previous studies for each disease. The administration of aspirin reduced atherosclerosis in ApoE KO mice [30, 31]. A cross-sectional study of the US population showed that patients with NAFLD who were regularly administered aspirin had a lower prevalence of NAFLD than those who did not receive aspirin [32]. Therefore, aspirin may systemically and simultaneously ameliorate NAFLD and atherosclerosis; furthermore, specific biomarkers for atherosclerosis can be expressed in the liver of patients with NAFLD, and it is feasible vice versa.

Many studies have reported the effects or roles of PPAR*δ*, AMPK, and PGC-1*α* on NAFLD and atherosclerosis. PPAR*δ* agonist, GW501516, treatment alleviated NAFLD induced in mice administered with high-fat diet and lipopolysaccharide [33]. Tert-butyl-4-(2-hydroxyethyl)-4-(pyrrolidin-1-yl)-piperidine-1-carboxylate (PYPEP), which is another PPAR*δ* agonist, inhibited atherosclerosis in human apolipoprotein B100 and cholesteryl ester transfer protein double-transgenic mice by improving the serum lipoprotein profiles [34]. Furthermore, the anti-NAFLD effects of dioscin and chicory (*Cichorium intybus* L.) polysaccharides were dependent to AMPK [35, 36], and in ApoE knock-out mice, AMPK activation by agonists decreased the formation of atheromata-inducing macrophages, but it increased the antiatherogenic effects of HDL-cholesterol [37, 38].

A disease-prone polymorphism of the PGC-1*α* gene was reported to elevate the risk of NAFLD in obese children [39], and lowered protein expression of PGC-1*α* was corelated with the development of atherosclerosis [40].

In addition to the direct inhibitory effects on NAFLD and atherosclerosis, PPAR*δ*, AMPK, and PGC-1*α* activate the catabolic metabolism in cells as a basal mechanism of anti-NAFLD and antiatherosclerosis: PPAR*δ* and AMPK activate oxidative phosphorylation and anti-inflammation, and PGC-1*α* is involved in mitochondrial biogenesis and increases oxidative phosphorylation [41–43]. In addition, PGC-1*α* expression is modulated by AMPK [44], and AMPK may be regulated by PPAR*δ* [45]. Therefore, the therapeutic functions for NAFLD and atherosclerosis of aspirin can be done through the increment of catabolic metabolism.

From blood analysis results in which aspirin administration lowered the levels of TG, ALT, and AST and increased HDL concentration, we can suppose the potential of aspirin as a medicine for atherosclerosis and NAFLD, and it was proved through additional cell and animal experiments. In our *in vitro* study, aspirin normalized the expression of biomarkers related to atherosclerosis and NAFLD in disease-mimicking conditions, and the effects of aspirin may be regulated by the PPAR*δ*-AMPK-PGC-1*α* pathway. Moreover, elevation of the oxygen consumption rate by aspirin suggests that aspirin can activate oxidative phosphorylation to produce ATP; this suggestion is strongly supported by the increased ATP levels in the aorta and liver tissues from the aspirin-administered rabbits. In addition, the ATP level-increasing effect of aspirin was strongly supported by the elevation of TFAM protein expression in the condition of aspirin treatment ([Supplementary-material supplementary-material-1]). TFAM as a transcription factor for mitochondrial DNA is a key role in the maintenance of mitochondrial DNA and restores ATP production decreased in hyperglycemic condition [46].

Generally, inflammation is involved in NAFLD and atherosclerosis [7]. In this study, aspirin decreased the levels of TNF*α* and lipid accumulation in HepG2 cells, and the effects of aspirin were reversed by PPAR*δ* antagonist. So the results supposed that aspirin can ameliorate atherosclerosis and NAFLD, and the effects of aspirin are dependent to PPAR*δ*.

Particularly notable results of the *in vitro* experiments were that aspirin lowered the level of AT1R in both HUVEC and HepG2 cells; therefore, aspirin may simultaneously affect atherosclerosis and NAFLD. Furthermore, our animal study demonstrated that AT1R and macrophage antigen proteins involved in the pathogenesis of atherosclerosis were simultaneously expressed in both the aorta and liver, and the expressions were modulated by aspirin. So these results suggest that atherosclerosis and NAFLD have a common pathogenesis.

The spleen is an important organ with the following essential functions: the filtering of blood to eliminate aged erythrocytes and unnecessary foreign substances; the exerting of the adoptive immune response that comprises antibody production; and the possessing of mononuclear phagocytes that include monocytes, macrophages, and dendritic cells [47, 48]. Therefore, the spleen reflects the state of the macrophages *in vivo*. In our study, the expression of CCR2, which is a macrophage-presenting marker involved in the onset of atherosclerosis and NAFLD, was significantly lower in the spleen tissues from aspirin-administered rabbits; however, the expression of mannose receptor, which represents an anti-inflammatory phenotype of macrophages, was markedly elevated in the spleen tissues from aspirin-administered rabbits. The *in vivo* results of CCR2 and mannose receptor agreed with the results of the RAW 264.7 macrophage cells. Therefore, atherosclerosis and NAFLD have a common pathology, and aspirin can prevent and ameliorate the two diseases at the same time.

The experiments of antagonists for PPAR*δ* and AMPK in HepG2 cells suggest that the protein expression of AMPK was regulated by PPAR*δ*. Likewise, in our previous HUVECs study, the protein level of AMPK was also regulated by PPAR*δ* [45].

## 5. Conclusion

**Figure 9:**
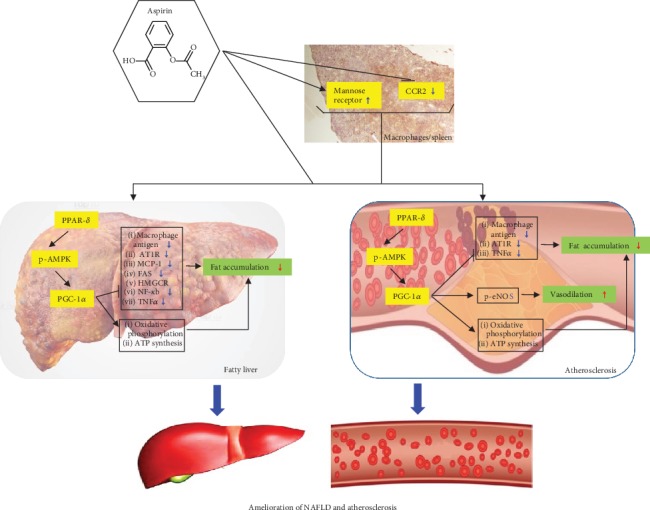
Hypothetical action mechanism of aspirin on nonalcoholic fatty liver and atherosclerosis. Simultaneous amelioration of both nonalcoholic fatty liver and atherosclerosis by aspirin is mainly achieved by catabolic activation, anti-inflammation, or vasodilation, which are the result of sequential regulation of the PPAR*δ*→p-AMPK→PGC-1*α*→oxidative phosphorylation pathway. In addition, the effects of aspirin are achieved by macrophage modulations, which include the increase of mannose receptor and decrease of CCR2.

In conclusion, aspirin can systemically and simultaneously ameliorate atherosclerosis and NAFLD through the following two routes: in the first main route, it activates catabolic lipid metabolism (decrease of lipid accumulation in HepG2 cells, the aorta, and the liver) and decreases inflammation (decreases of NF-*κ*B and TNF*α*), and these two reactions are mediated through the PPAR*δ*-AMPK-PGC-1*α* pathway (the effects of aspirin on lipid catabolism and inflammation are mainly regulated by PPAR*δ*), and in the second subsidiary route, it downregulates CCR2 and induces anti-inflammatory phenotype in macrophages (Figure 9). The positive functions of aspirin may be also supported through the amelioration effects on the decreased diet intake amount and weight loss by cholesterol diet ([Supplementary-material supplementary-material-1]). A unique advantage of our study is the first systemic and integrated research of aspirin for atherosclerosis and NAFLD. Therefore, our research can offer a useful tool or paradigm to develop newly efficient drugs simultaneously acting on NAFLD and atherosclerosis. Because our animal study was deficient parts in metabolic experiments, it will be compensated in the following study.

## Figures and Tables

**Table 1:** Ingredients of normal control and cholesterol diets.

Normal control diet composition		
Nutrients		
Protein	%	16.50
Arginine	%	0.97
Cystine	%	0.29
Glycine	%	0.73
Histidine	%	0.33
Isoleucine	%	0.66
Leucine	%	1.20
Lysine	%	0.80
Methionine	%	0.24
Phenylalanine	%	0.75
Tyrosine	%	0.49
Threonine	%	0.63
Tryptophane	%	0.23
Valine	%	0.80
Fat (ether extract)	%	2.47
Linoleic acid	%	1.15
Linolenic acid	%	0.29
Arachidonic acid	%	0.02
Omega-3 fatty acids	%	1.17
Fiber (crude)	%	16.72
Minerals		
Ash	%	9.06
Calcium	%	1.40
Phosphorus	%	0.50
Phosphorus (nonphytate)	%	0.23
Potassium	%	1.31
Magnesium	%	0.27
Sulfur	%	0.21
Sodium	%	0.34
Chlorine	%	0.56
Fluorine	ppm	14.69
Iron	ppm	298.28
Zinc	ppm	158.52
Manganese	ppm	49.71
Copper	ppm	41.35
Cobalt	ppm	1.22
Iodine	ppm	2.12
Chromium	ppm	0.00
Selenium	ppm	0.66
Vitamins		
Vitamin K	ppm	4.80
Thiamin hydrochloride	ppm	7.07
Riboflavin	ppm	9.78
Niacin	ppm	70.48
Pantothenic acid	ppm	34.16
Choline chloride	ppm	1300.00
Folic acid	ppm	4.60
Pyridoxine	ppm	8.31
Biotin	ppm	0.23
Vitamin B12	ppm	10.00
Vitamin A	IU/g	28.89
Vitamin D3 (added)	IU/g	1.00
Vitamin E	IU/kg	73.92
Calories provided by:		
Protein	%	25.808
Fat (ether extract)	%	8.690
Carbohydrates	%	65.502

^∗^Nutrients expressed as percent of ration except where otherwise indicated. Moisture content is assumed to be 10.0% for the purpose of calculations. ^#^The cholesterol diet was made by adding 1% cholesterol to normal control diet as a ratio of weight/weight.

**Table 2:** Cholesterol diet composition.

Ingredient	mg
Normal control diet	990
Cholesterol	10
Total	1000

## Data Availability

In sharing options for data availability, we can provide the data when there is a request from other researcher (Data Available on Request). The e-mail address for the data request is as follows; mdhsseo@unitel.co.kr and lyj2333@hanmail.net.

## Supplementary Materials

Supplementary Materials Supplementary Figure 1: The mean amount of diet intake (A) per rabbit per week and the mean body weight (B) per rabbit perweek. Supplementary Figure 2: Western blot analysis (A) and ELISA (B) of TFAM in HepG2 cells that were treated with high concentrations of palmitate and cholesterol, aspirin, and GSK0660.(Supplementary materials)Supplementary Table. The correlation analysis among macrophage antigen, AT1R, and Oil Red O staining in liver and aorta from rabbits.

## References

[1] Villanova N., Moscatiello S., Ramilli S. (2005). Endothelial dysfunction and cardiovascular risk profile in nonalcoholic fatty liver disease. *Hepatology*.

[2] Sookoian S., Pirola C. J. (2008). Non-alcoholic fatty liver disease is strongly associated with carotid atherosclerosis: a systematic review. *Journal of Hepatology*.

[3] Targher G., Day C. P., Bonora E. (2010). Risk of cardiovascular disease in patients with nonalcoholic fatty liver disease. *The New England Journal of Medicine*.

[4] Gastaldelli A., Kozakova M., Højlund K. (2009). Fatty liver is associated with insulin resistance, risk of coronary heart disease, and early atherosclerosis in a large European population. *Hepatology*.

[5] Tuzcu E. M., Kapadia S. R., Tutar E. (2001). High prevalence of coronary atherosclerosis in asymptomatic teenagers and young adults: evidence from intravascular ultrasound. *Circulation*.

[6] Kleemann R., Verschuren L., van Erk M. J. (2007). Atherosclerosis and liver inflammation induced by increased dietary cholesterol intake: a combined transcriptomics and metabolomics analysis. *Genome Biology*.

[7] Kim E. J., Kim B. H., Seo H. S. (2014). Cholesterol-induced non-alcoholic fatty liver disease and atherosclerosis aggravated by systemic inflammation. *PLoS One*.

[8] Meade E. A., Smith W. L., DeWitt D. (1993). Differential inhibition of prostaglandin endoperoxide synthase (cyclooxygenase) isozymes by aspirin and other non-steroidal anti-inflammatory drugs. *The Journal of Biological Chemistry*.

[9] Berger J., Moller D. E. (2002). The mechanisms of action of PPARs. *Annual Review of Medicine*.

[10] Feige J. N., Gelman L., Michalik L., Desvergne B., Wahli W. (2006). From molecular action to physiological outputs: peroxisome proliferator-activated receptors are nuclear receptors at the crossroads of key cellular functions. *Progress in Lipid Research*.

[11] Wan J., Jiang L., Lü Q., Ke L., Li X., Tong N. (2010). Activation of PPARdelta up-regulates fatty acid oxidation and energy uncoupling genes of mitochondria and reduces palmitate-induced apoptosis in pancreatic beta-cells. *Biochemical and Biophysical Research Communications*.

[12] Hardie D. G., Ross F. A., Hawley S. A. (2012). AMPK: a nutrient and energy sensor that maintains energy homeostasis. *Nature Reviews Molecular Cell Biology*.

[13] Hardie D. G. (2013). AMPK: a target for drugs and natural products with effects on both diabetes and cancer. *Diabetes*.

[14] Kim S. J., Tang T., Abbott M., Viscarra J. A., Wang Y., Sul H. S. (2016). AMPK phosphorylates desnutrin/ATGL and hormone-sensitive lipase to regulate lipolysis and fatty acid oxidation within adipose tissue. *Molecular and Cellular Biology*.

[15] Lehman J. J., Barger P. M., Kovacs A., Saffitz J. E., Medeiros D. M., Kelly D. P. (2000). Peroxisome proliferator-activated receptor gamma coactivator-1 promotes cardiac mitochondrial biogenesis. *The Journal of Clinical Investigation*.

[16] Olesen J., Kiilerich K., Pilegaard H. (2010). PGC-1alpha-mediated adaptations in skeletal muscle. *Pflügers Archiv*.

[17] Wennmalm A. (1994). Nitric oxide (NO) in the cardiovascular system: role in atherosclerosis and hypercholesterolemia. *Blood Pressure*.

[18] Na L., Chu X., Jiang S. (2016). Vinegar decreases blood pressure by down-regulating AT1R expression via the AMPK/PGC-1*α*/PPAR*γ* pathway in spontaneously hypertensive rats. *European Journal of Nutrition*.

[19] Hernández J. S., Barreto-Torres G., Kuznetsov A. V., Khuchua Z., Javadov S. (2014). Crosstalk between AMPK activation and angiotensin II-induced hypertrophy in cardiomyocytes: the role of mitochondria. *Journal of Cellular and Molecular Medicine*.

[20] Sun H., Zhu X., Zhou Y., Cai W., Qiu L. (2017). C1q/TNF-related protein-9 ameliorates ox-LDL-induced endothelial dysfunction via PGC-1*α*/AMPK-mediated antioxidant enzyme induction. *International Journal of Molecular Sciences*.

[21] Videla L. A., Rodrigo R., Araya J., Poniachik J. (2006). Insulin resistance and oxidative stress interdependency in non-alcoholic fatty liver disease. *Trends in Molecular Medicine*.

[22] Kim J. H., Na H. J., Kim C. K. (2008). The non-provitamin A carotenoid, lutein, inhibits NF-*κ*B-dependent gene expression through redox-based regulation of the phosphatidylinositol 3-kinase/PTEN/Akt and NF-*κ*B-inducing kinase pathways: Role of H_2_O_2_ in NF-*κ*B activation. *Free Radical Biology and Medicine*.

[23] Miura K., Yang L., van Rooijen N., Ohnishi H., Seki E. (2012). Hepatic recruitment of macrophages promotes nonalcoholic steatohepatitis through CCR2. *American Journal of Physiology-Gastrointestinal and Liver Physiology*.

[24] Boring L., Gosling J., Cleary M., Charo I. F. (1998). Decreased lesion formation in CCR2-/- mice reveals a role for chemokines in the initiation of atherosclerosis. *Nature*.

[25] Mills C. (2012). M1 and M2 macrophages: oracles of health and disease. *Critical Reviews in Immunology*.

[26] Wan J., Benkdane M., Teixeira-Clerc F. (2014). M2 Kupffer cells promote M1 Kupffer cell apoptosis: a protective mechanism against alcoholic and nonalcoholic fatty liver disease. *Hepatology*.

[27] Aiello R. J., Bourassa P. A. K., Lindsey S. (1999). Monocyte chemoattractant protein-1 accelerates atherosclerosis in apolipoprotein E-deficient mice. *Arteriosclerosis, Thrombosis, and Vascular Biology*.

[28] Kleinbongard P., Heusch G., Schulz R. (2010). TNFalpha in atherosclerosis, myocardial ischemia/reperfusion and heart failure. *Pharmacology & Therapeutics*.

[29] Yang B. C., Phillips M. I., Mohuczy D. (1998). Increased angiotensin II type 1 receptor expression in hypercholesterolemic atherosclerosis in rabbits. *Arteriosclerosis, Thrombosis, and Vascular Biology*.

[30] Kraus S., Naumov I., Shapira S. (2014). Aspirin but not meloxicam attenuates early atherosclerosis in apolipoprotein E knockout mice. *The Israel Medical Association Journal*.

[31] Sorokin A. V., Yang Z. H., Vaisman B. L. (2016). Addition of aspirin to a fish oil-rich diet decreases inflammation and atherosclerosis in ApoE-null mice. *The Journal of Nutritional Biochemistry*.

[32] Shen H., Shahzad G., Jawairia M., Bostick R. M., Mustacchia P. (2014). Association between aspirin use and the prevalence of nonalcoholic fatty liver disease: a cross-sectional study from the Third National Health and Nutrition Examination Survey. *Alimentary Pharmacology & Therapeutics*.

[33] Lee H. J., Yeon J. E., Ko E. J. (2015). Peroxisome proliferator-activated receptor-delta agonist ameliorated inflammasome activation in nonalcoholic fatty liver disease. *World Journal of Gastroenterology*.

[34] Naya N., Fukao K., Nakamura A. (2016). A selective peroxisome proliferator-activated receptor *δ* agonist PYPEP suppresses atherosclerosis in association with improvement of the serum lipoprotein profiles in human apolipoprotein B100 and cholesteryl ester transfer protein double transgenic mice. *Metabolism*.

[35] Yao H., Tao X., Xu L. (2018). Dioscin alleviates non-alcoholic fatty liver disease through adjusting lipid metabolism via SIRT1/AMPK signaling pathway. *Pharmacological Research*.

[36] Wu Y., Zhou F., Jiang H., Wang Z., Hua C., Zhang Y. (2018). Chicory (*Cichorium intybus* L.) polysaccharides attenuate high-fat diet induced non-alcoholic fatty liver disease via AMPK activation. *International Journal of Biological Macromolecules*.

[37] Wang J., Ma A., Zhao M., Zhu H. (2017). AMPK activation reduces the number of atheromata macrophages in ApoE deficient mice. *Atherosclerosis*.

[38] Ma A., Wang J., Yang L., An Y., Zhu H. (2017). AMPK activation enhances the anti-atherogenic effects of high density lipoproteins in apoE-/- mice. *Journal of Lipid Research*.

[39] Lin Y. C., Chang P. F., Chang M. H., Ni Y. H. (2013). A common variant in the peroxisome proliferator-activated receptor-*γ* coactivator-1*α* gene is associated with nonalcoholic fatty liver disease in obese children. *The American Journal of Clinical Nutrition*.

[40] Valero-Muñoz M., Martín-Fernández B., Ballesteros S. (2013). Relevance of vascular peroxisome proliferator-activated receptor *γ* coactivator-1*α* to molecular alterations in atherosclerosis. *Experimental Physiology*.

[41] Röckl K. S., Hirshman M. F., Brandauer J., Fujii N., Witters L. A., Goodyear L. J. (2007). Skeletal muscle adaptation to exercise training: AMP-activated protein kinase mediates muscle fiber type shift. *Diabetes*.

[42] Narkar V. A., Downes M., Yu R. T. (2008). AMPK and PPAR*δ* Agonists Are Exercise Mimetics. *Cell*.

[43] LeBleu V. S., O’Connell J. T., Gonzalez Herrera K. N. (2014). PGC-1*α* mediates mitochondrial biogenesis and oxidative phosphorylation in cancer cells to promote metastasis. *Nature Cell Biology*.

[44] Wan Z., Root-McCaig J., Castellani L., Kemp B. E., Steinberg G. R., Wright D. C. (2014). Evidence for the role of AMPK in regulating PGC-1 alpha expression and mitochondrial proteins in mouse epididymal adipose tissue. *Obesity (Silver Spring)*.

[45] Lee Y. J., Jang Y. N., Han Y. M., Kim H. M., Seo H. S. (2018). 6-Gingerol normalizes the expression of biomarkers related to hypertension via PPAR*δ* in HUVECs, HEK293, and differentiated 3T3-L1 cells. *PPAR Research*.

[46] Suarez J., Hu Y., Makino A., Fricovsky E., Wang H., Dillmann W. H. (2008). Alterations in mitochondrial function and cytosolic calcium induced by hyperglycemia are restored by mitochondrial transcription factor a in cardiomyocytes. *American Journal of Physiology. Cell Physiology*.

[47] Bronte V., Pittet M. J. (2013). The spleen in local and systemic regulation of immunity. *Immunity*.

[48] Swirski F. K., Nahrendorf M., Etzrodt M. (2009). Identification of splenic reservoir monocytes and their deployment to inflammatory sites. *Science*.

