# Supplementary material for: Aspirin Improves Nonalcoholic Fatty Liver Disease and Atherosclerosis through Regulation of the PPARδ-AMPK-PGC-1α Pathway in Dyslipidemic Conditions
Source: Biomed Res Int. 2020 Mar 19;2020:7806860. doi: 10.1155/2020/7806860 (PMC7106881; doi:10.1155/2020/7806860)
Supplement: Supplementary Materials — Supplementary Figure 1: The mean amount of diet intake (A) per rabbit per week and the mean body weight (B) per rabbit perweek. Supplementary Figure 2: Western blot analysis (A) and ELISA (B) of TFAM in HepG2 cells that were treated with high concentrations of palmitate and cholesterol, aspirin, and GSK0660.(Supplementary materials)Supplementary Table. The correlation analysis among macrophage antigen, AT1R, and Oil Red O staining in liver and aorta from rabbits. [file 7806860.f1.pdf]

Supplementary figure legends

Supplementary Figure. 1 The mean amount of diet intake (A) per rabbit per week and the mean body weight (B) per rabbit per week.

The diet intake amount and body weight decreased by cholesterol diet were ameliorated via the treatment of aspirin.

Supplementary Figure. 2 Western blot analysis (A) and ELISA (B) of TFAM in HepG2 cells that were treated with high concentrations of palmitate and cholesterol, aspirin, and GSK0660.

In Western blotting and ELISA results, the decreased TFAM protein levels in CP group compared to control were increased in CPA group, however, the effect of aspirin was reversed by PPARδ antagonist, GSK0660. Values were statistically analyzed by unpaired t-test and one way ANOVA. An upper line on the three bars means one way ANOVA analysis. All experiments were over repeated three and over times. Meaning of indications: Ctrl is an untreated control group, CP is a cholesterol and palmitate treated group, CPA is a cholesterol, palmitate, and aspirin treated group, and CPAG is a cholesterol, palmitate, aspirin, and GSK0660 treated group. * $p < 0.05$, ** $p < 0.01$, *** $p < 0.001$.

Supplementary figure. 1

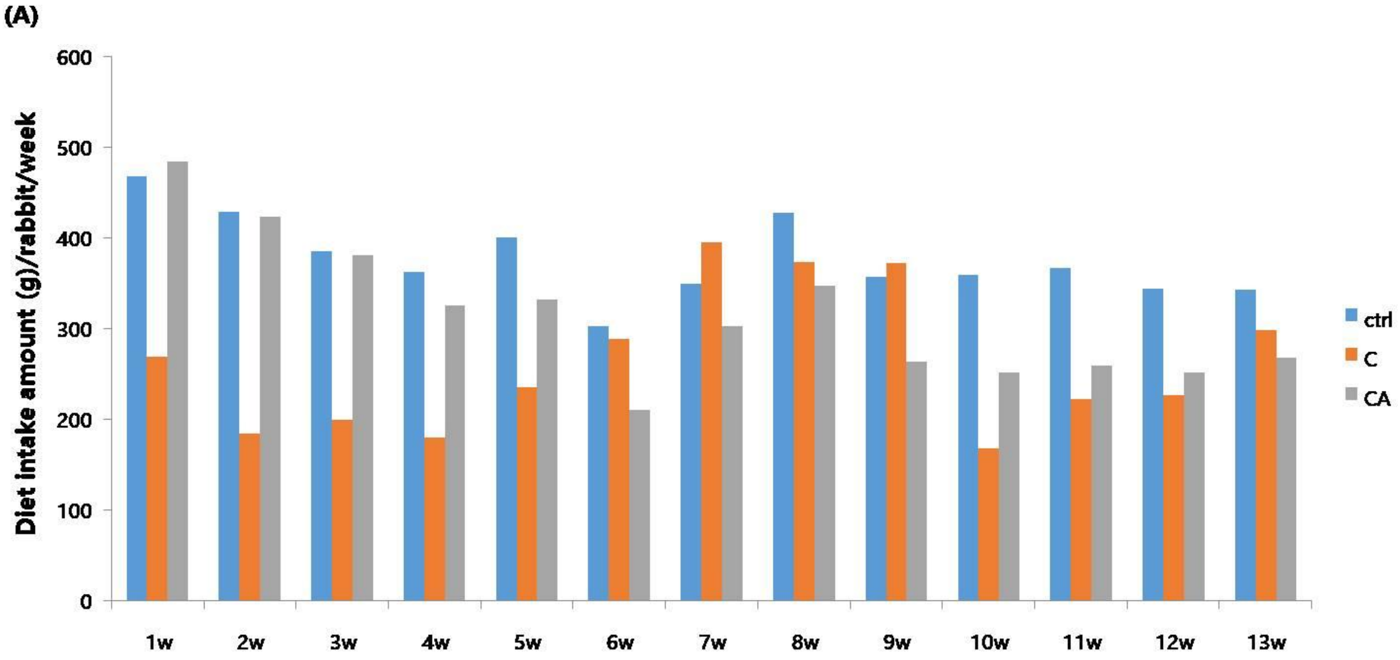

(B)

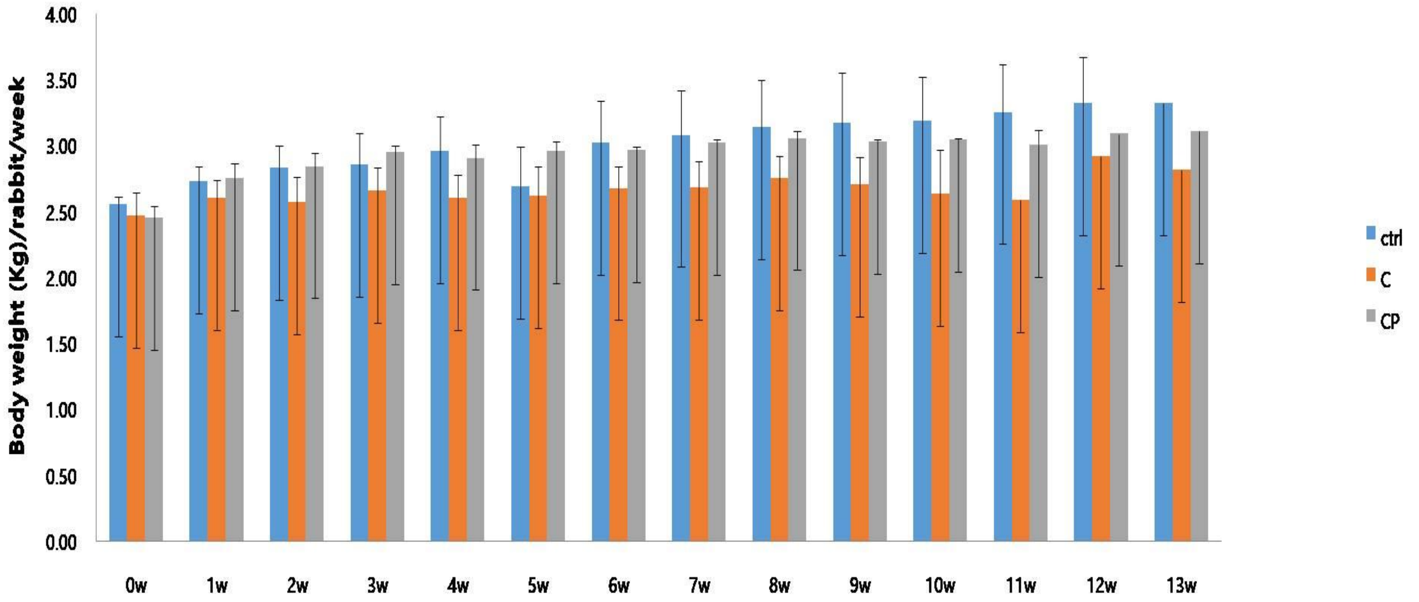
4.00
3.50
3.00
2.50
2.00
1.50
1.00
0.50
0.00
Body weight (Kg)/rabbit/week
0w
1w
2w
3w
4w
5w
6w
7w
8w
9w
10w
11w
12w
13w
ctrl
C
CP

Supplementary figure. 2

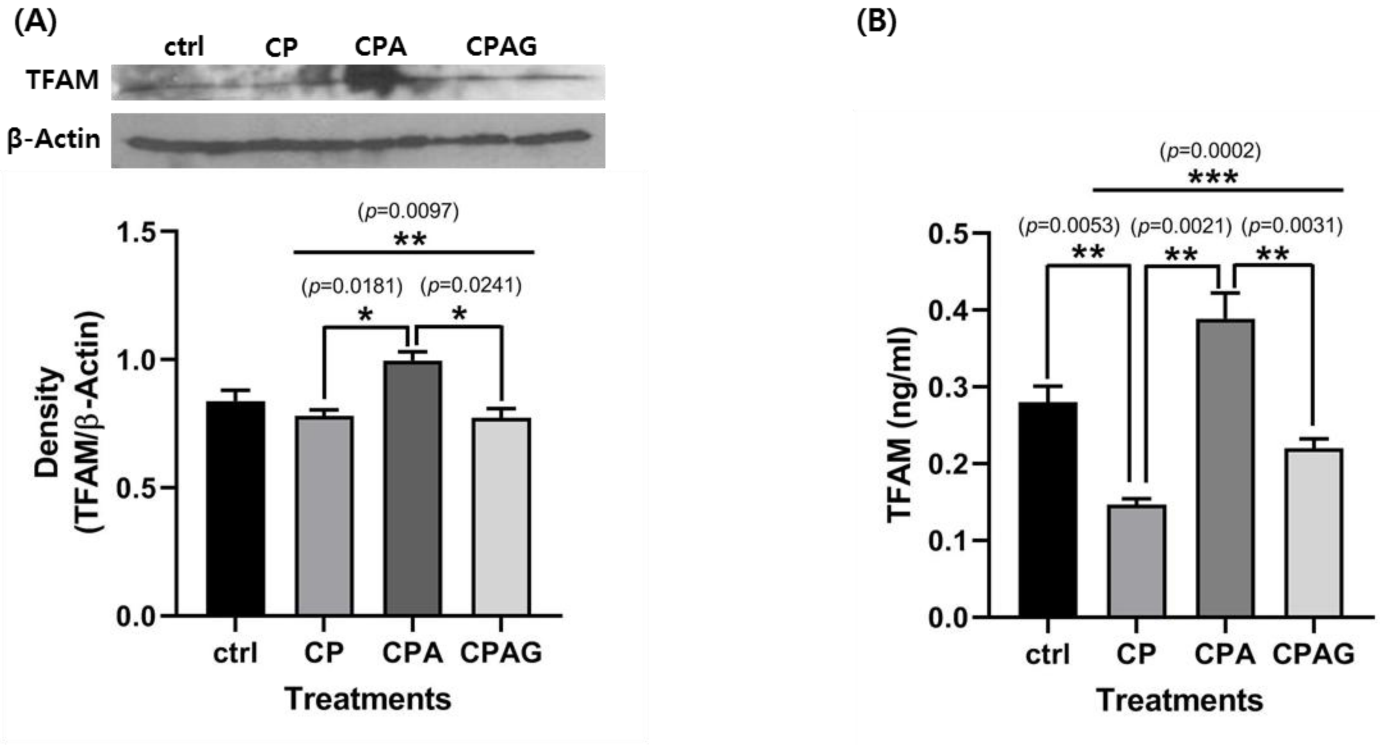

Supplementary Table. The correlation analysis among macrophage antigen, AT1R, and Oil Red O staining in liver and aorta from rabbits

Correlations

			liver macrophage antigen IHC	liver AT1R IHC	liver OIL R O	aorta macrophage antigen IHC	aorta AT1R IHC	aorta OIL R O
Spearman's rho	liver macrophage antigen IHC	Correlation Coefficient	1.000	.525*	.441	.313	.450*	.340
		Sig. (2-tailed)	.	.018	.052	.179	.047	.143
		N	20	20	20	20	20	20
	liver AT1R IHC	Correlation Coefficient	.525*	1.000	.693**	.756**	.809**	.600**
		Sig. (2-tailed)	.018	.	.001	.000	.000	.005
		N	20	20	20	20	20	20
	liver OIL R O	Correlation Coefficient	.441	.693**	1.000	.832**	.750**	.797**
		Sig. (2-tailed)	.052	.001	.	.000	.000	.000
		N	20	20	20	20	20	20
	aorta macrophage antigen IHC	Correlation Coefficient	.313	.756**	.832**	1.000	.792**	.866**
		Sig. (2-tailed)	.179	.000	.000	.	.000	.000
		N	20	20	20	20	20	20
	aorta AT1R IHC	Correlation Coefficient	.450*	.809**	.750**	.792**	1.000	.713**
		Sig. (2-tailed)	.047	.000	.000	.000	.	.000
		N	20	20	20	20	20	20
	aorta OIL R O	Correlation Coefficient	.340	.600**	.797**	.866**	.713**	1.000
		Sig. (2-tailed)	.143	.005	.000	.000	.000	.
		N	20	20	20	20	20	20

*. Correlation is significant at the 0.05 level (2-tailed).

**. Correlation is significant at the 0.01 level (2-tailed).
